# A Geospatial Bibliometric Review of the HIV/AIDS Epidemic in the Russian Federation

**DOI:** 10.3389/fpubh.2020.00075

**Published:** 2020-04-02

**Authors:** Megan E. Gray Neils, Herman O. I. Pfaeffle, Art T. Kulatti, Alena Titova, Galina S. Lyles, Yulia Plotnikova, Elena Zorkaltseva, Oleg B. Ogarkov, Serhiy M. Vitko, Rebecca A. Dillingham, Scott K. Heysell

**Affiliations:** ^1^UVA Division of Infectious Diseases & International Health, University of Virginia Medical Center, Charlottesville, VA, United States; ^2^University of Virginia School of Medicine, Charlottesville, VA, United States; ^3^College and Graduate School of Arts & Sciences, University of Virginia, Charlottesville, VA, United States; ^4^UVA Division of Pulmonary & Critical Care Medicine, University of Virginia Medical Center, Charlottesville, VA, United States; ^5^Irkutsk AIDS Center, Irkutsk, Russia; ^6^Irkutsk State Medical Postgraduate Education Academy, Irkutsk, Russia; ^7^Scientific Centre of the Family Health and Human Reproductive Problems, Irkutsk, Russia

**Keywords:** HIV, acquired immunodeficiency syndrome, Russia, USSR, bibliometrics, intravenous substance abuse, publications, tuberculosis

## Abstract

**Background:** Increasing rates of HIV/AIDS in Eastern Europe and Central Asia contrast global trends, but the scope of HIV/AIDS research originating from Russian Federation and countries of the former Soviet Union has not been quantified.

**Methods:** We searched six major scientific databases in Russian and English languages with medical subject heading terms “HIV” or “AIDS” and “Russia” or “Soviet Union” from 1991 to 2016. Each abstract indexed was reviewed and tagged for 25 HIV/AIDS research themes, location of research focus and first author.

**Results and Discussion:** A total of 2,868 articles were included; 2,156 (75.1%) and 712 (24.8%) described research in the Russian Federation and countries of the former Soviet Union, respectively. There were 15 publications per million population in Russian Federation. Federal districts of the Russian Federation with the highest rates of HIV had the most limited publications. An interactive web-map with time-lapse features and links to primary literature was created using ArcGIS^®^ technology [http://arcg.is/2FUIJ5v].

**Conclusion:** We found a lower than expected publication rate in the Russian Federation relative to rising HIV prevalence. The greatest deficits were in the most HIV burdened regions in the Russian Federation. Our findings highlight opportunities for new research strategies and public health efforts among key populations and subnational regions.

## Introduction

There are increasing rates of new Human Immunodeficiency Virus (HIV) infections in Eastern Europe and Central Asia, while the majority of other regions of the world now have decreasing rates of infection (1). The Russian Federation estimates over 1.1 million people living with HIV in the country in 2017 accounting for 80% of new HIV infections in all of Eastern Europe and Central Asia (2, 3). Research is required to inform appropriate scientific, public health, and policy interventions to combat the HIV epidemic and published output is generally reflective of near-current federal or regional priorities. As HIV research and public health efforts are expanding across the world, the extent and scope of HIV research in the Russian Federation and Eastern Europe, mainly consisting of the countries of the former Soviet Union, have not yet be quantified or comparatively examined.

The Soviet Union dissolved in 1991. The first case of HIV was seen 4 years prior (4). This political and legislative transition led to significant economic and social challenges for both the Russian Federation and countries of the former Soviet Union. Though the Russian Federation is now considered an upper middle-income country, the response to the HIV epidemic has differed from other higher income settings (5). Currently, new HIV infections in the Russian Federation are acquired through injection drug use (IDU) in half of cases. However, the epidemic is growing not only in higher-risk populations such as people who inject drugs (PWID), men who have sex with men (MSM), and sex workers, but also in the those less historically marginalized from the health care system (2). As of 2016, HIV was one of the top ten causes of premature death in the Russian Federation, a 41% proportional increase from 2005 (6). The progression of HIV infection to acquired immunodeficiency syndrome (AIDS) and co-infections with Hepatitis B virus, Hepatitis C virus (HCV), and *Mycobacterium tuberculosis* (TB) all contribute to the rising mortality rate (7).

In 2015 the Russian Government released a “State Strategy to Combat the Spread of HIV” with goals to expand HIV screening, increase antiretroviral availability and reduce new HIV infections as well as mortality related to AIDS and co-morbid infections, specifically viral hepatitis and TB (7). HCV co-infection in people living with HIV is known to lead to more rapid progression to decompensated cirrhosis (8). Seventy-two percent of PWID in the Russian Federation are infected with HCV (9) and treatment for HCV is not routinely available to PWID (10, 11). TB has been a major public health issue in the Russian Federation since the early twentieth century (12), but contemporary Russia now faces the additional challenge of multi-drug resistant TB (MDR-TB). Thirty-four percent of all cases of TB in the Russian Federation are MDR-TB making it the third-highest burdened country in the world (13). PWID are the highest risk group for the acquisition of TB and MDR-TB in the Russian Federation, and co-infection with HIV increases treatment failure 7-fold and risk of death 3-fold (14). Eastern Europe also confronts similar challenges with MDR-TB (15). However, each country of the former Soviet Union has unique political and social landscapes that make it unwise to consider Eastern Europe and Central Asia as a distinct epidemiologic expanse without more localized data.

Given the immense HIV epidemic in the region of the Russian Federation and countries of the former Soviet Union, a robust research response would be expected. The first bibliometric analysis of AIDS was published in 1992, and captured the scientific literature that presented HIV as the causative infection of AIDS (4). Since that time, several bibliometric analyses have been completed to evaluate HIV/AIDS research in specific regions of the world (16–21), none have analyzed HIV/AIDS literature specifically in the Russian Federation. In order to better understand how the HIV epidemic in this region has been assessed and measured, we aimed to capture publicly available scientific literature pertaining to HIV/AIDS in the Russian Federation and countries of the former Soviet Union in both English and Russian language databases. Understanding the limitations of causative assumptions from this bibliometric work, we further developed an interactive web-map that could be used by researchers and policy makers to guide future interventions and allow for additional evaluation of research output.

## Methods

### Bibliometric Analysis Study Design

Four major English-based scientific databases: PubMed^®^, Web of Science^®^, Embase (Elsevier), and eLibrary^®^, and two additional Russian databases: Central Scientific Medical Library, and Moscow State Medical University Library were searched using the medical subject heading terms “HIV” or “AIDS” AND “Russia” or “Soviet Union”- a term that links to other country-specific headings. The search was limited to articles electronically published after December 31, 1990 to coincide with the dissolution of the Soviet Union and the beginning of the HIV epidemic in the region, and before January 1, 2017 to assure to account for delayed postings of publications in the final year of review. Articles in English or Russian were included if HIV/AIDS, Russian Federation and/or former Soviet Union were contained within the title or abstract text, or if any author locations were in the Russian Federation or countries of the former Soviet Union. News articles, editorials, commentaries, and those that did not fit the search criteria were excluded.

### Data Collection and Analysis

Title, author details and the available abstract text from each article were reviewed. Each article was manually tagged for 25 HIV research themes based on the information presented in the title and abstract. The city and country of the research focus were also collected. The research focus either represented the population of interest or the location where the laboratory research was conducted. In addition, the location of the first author was collected. Articles and data were initially stored using Zotero citation manager^®^, which also automatically extracted certain bibliographic data, such as article title, abstract text, author information, and digital object identifiers from included articles. Microsoft Excel^®^ was used for data exportation and cleaning before transfer to IBM SPSS Statistics^®^, which was used for analysis. The chi-squared test was used to evaluate the differences in research themes with Bonferroni correction to determine statistical significance of multiple comparisons.

### Web-Map Development

Data was translated into an interactive web application using ArcGIS^®^. Within the application there are three main maps. Map one displays the quantity of research articles from the Russian Federation over the geographic region from which the data or research topic originated. Map two displays the quantity of published studies based on the location of the primary researcher, or first author, highlighting potential research partnerships across the world. Map three and four are time-lapse maps of Map one and two respectively, that visually depict the progression of research from 1991 to 2016. Map one and two have a filter function that allows for interactive visualization as users adjust date ranges and narrow the map results by research themes or location. Additionally, article details are displayed below maps with hyperlinks to full article texts if available on public domains. Tables presenting analyzed data are also displayed below the first two maps. This web application is available to the public: [http://arcg.is/2FUIJ5v].

### Thematic Analysis

A core group of authors collectively enumerated the major findings of the comparative analysis for the Russian Federation to countries of the former Soviet Union and regional differences within the Russian Federation. Thematic analyses were centered in major priorities of international consensus statements for ending the HIV epidemic and the policy priorities of the Russian government's “Strategy to Combat the Spread of HIV.” Prioritized themes were displayed visually in Results figure and included in the Discussion.

## Results

**Figure 1:**
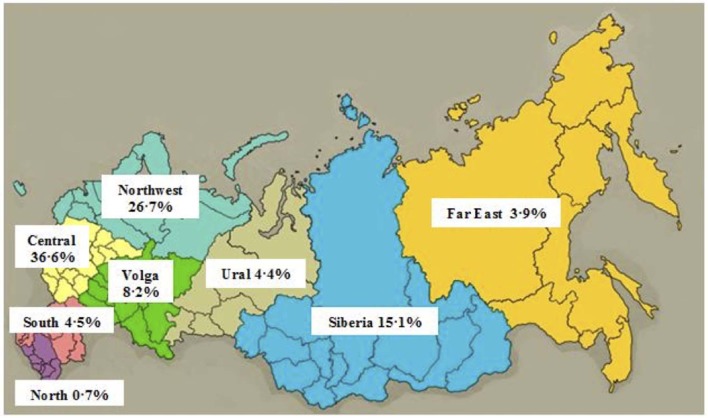
Percentage of total research articles originating from each federal district of Russia. ^*^Percentages calculated from the total articles with known locations in Russian Federal Districts (i.e., Russia-All, Russia-Unknown are excluded).

A total of 2,868 articles were included, 2,156 (75.1%) and 712 (24.8%) had research focus in the Russian Federation and countries of the former Soviet Union, respectively. Of the 2,156 articles originating from the Russia, 713 (33.9%) had no clear location of the research focus. They were categorized as originating either from “Russia—ALL,” indicating that there was more than one federal district included (177, 7.2%), or “Russia—UNKNOWN,” indicating that no specific city or federal district was included (554, 25.7%) (Table 1 and Figure 1).

**Table 1:** Quantity of research articles per Russian federal district separated by research theme from 1991 to 2016.

	**Total**	**North Caucasus**	**Southern**	**Central**	**Volga**	**North-West**	**Ural**	**Siberian**	**Far East**	**Russia - All**	**Russia - Unknown**
Number of articles	2,156	10	64	521	117	380	63	215	55	177	554
**Research Theme**	(% of Districts)	0.7	4.5	36.6	8.2	26.7	4.4	15.1	3.9	–	–
	(% of Total)	0.5	3	24.2	5.4	17.6	2.9	10	2.6	8.2	25.7
Injection drug use	544	6	24	81	33	167	19	46	6	61	101
Virology	717	3	28	252	30	50	26	128	39	27	134
Sexually transmitted diseases	477	0	6	59	21	126	26	48	36	22	133
Prevention	394	0	16	65	23	97	6	38	0	50	99
Genetics/genomics	427	3	13	181	15	30	9	83	11	11	71
Antiretroviral therapy	319	2	7	102	20	48	12	26	3	23	76
Policy	267	0	3	29	13	60	7	3	0	70	82
Infants children adolescents	194	4	13	29	9	38	7	12	1	16	65
Tuberculosis	211	0	3	31	28	28	5	23	4	27	62
Substance use disorder	185	1	6	28	9	68	5	9	0	15	44
Opportunistic infections	187	0	3	33	19	30	3	15	0	12	72
Access to care	130	0	1	13	7	49	6	4	0	18	32
Hepatitis C virus co-infection	141	1	3	23	12	25	6	16	0	13	42
Stigma	135	0	3	21	4	38	4	5	0	23	37
Sex workers	104	0	1	24	7	29	1	8	0	10	24
Alcohol	135	0	1	14	5	67	2	11	0	7	28
Males who have sex with males	106	2	5	23	2	34	4	3	0	9	24
Prisoners/incarceration	75	0	1	11	9	18	4	6	0	6	20
Medication adherence	51	0	1	5	4	23	1	1	0	3	13
Psychiatric illness	66	0	1	11	2	29	1	2	0	1	19
Pre-exposure prophylaxis	43	0	1	5	3	6	2	12	0	3	11
Retention in care	34	0	0	4	0	21	0	0	0	3	6
Coping/spirituality	19	0	2	2	1	8	1	1	0	1	3
Disclosure	18	0	0	1	0	13	0	0	0	0	4
Mobile health	5	0	0	1	0	2	0	0	0	1	1

*Research theme order based on total dataset*.

The most prolific countries of the former Soviet Union were the Ukraine and Estonia, publishing 29.2% and 14.3% of the total articles from all countries of the former Soviet Union (Supplemental Table 1 and Supplemental Figure 1).

The most common research themes were injection drug use, virology, and sexually transmitted infections. There were statistically significant differences in research themes from articles originating from the Russian Federation and from countries of the former Soviet Union, specifically injection drug use (25.2 vs. 48.6%, *p* = 0.001), access to care (6.0 vs. 13.8%, *p* = 0.001), and virology (33.3 vs. 15%, *p* = 0.001). There were unexpectedly low numbers of articles with TB as a research theme in the Russian Federation and former Soviet Union countries, 10.8 and 13.9% respectively (Table 2).

**Table 2:** A comparison of publication quantity and proportion by research theme relative to total output between the Russian Federation and countries of the Former Soviet Union from 1991 to 2016.

**Research theme**	***N* Total**	**% Total**	***N* RF**	**% RF**	***N* FSU**	**% FSU**	P*
Total articles	2868	100	2156	75.2	710	24.8	–
Injection drug use	890	31.0	544	25.2	346	48.6	**<0.001**
Virology	824	28.7	717	33.3	107	15	**<0.001**
Sexually transmitted diseases	640	22.3	477	22.1	163	22.9	0.678
Prevention	545	19.0	394	18.3	151	21.2	0.088
Genetics/genomics	544	19.0	427	19.8	117	16.4	0.047
Antiretroviral therapy	406	14.2	319	14.8	87	12.2	0.094
Policy	399	13.9	267	12.4	132	18.5	**<0.001**
Infants children adolescents	312	10.0	194	9.0	188	16.6	**<0.001**
Tuberculosis	310	10.8	211	9.8	99	13.9	0.003
Substance use disorder	291	10.1	185	8.6	106	14.9	**<0.001**
Opportunistic infections	278	9.7	187	8.7	91	12.8	0.002
Access to care	228	7.9	130	6.0	98	13.8	**<0.001**
Hepatitis C virus co-infection	212	7.4	141	6.5	71	10	0.004
Stigma	197	6.9	135	6.3	62	8.7	0.032
Sex workers	170	5.9	104	4.8	66	9.3	**<0.001**
Alcohol	163	5.7	135	6.3	28	3.9	0.019
Males who have sex with males	158	5.5	10.6	4.9	52	7.3	0.018
Prisoners/incarceration	113	3.9	75	3.5	38	5.3	0.034
Medication adherence	74	2.6	51	2.4	23	3.2	0.22
Psychiatric illness	72	2.5	66	3.1	6	0.8	**<0.001**
Pre-exposure prophylaxis	54	1.9	43	2.0	11	1.5	0.526
Retention in care	39	1.4	34	1.6	5	0.7	0.093
Coping/Spirituality	23	0.8	19	0.9	4	0.6	0.627
Disclosure	24	0.8	18	0.8	6	0.8	–
Mobile health	6	0.2	5	0.2	1	0.1	–

*P < 0.0021 (Bonferroni Correction Factor α = 0.05), RF, Russian Federation; FSU, Countries of the former Soviet Union*.

**Figure 2:**
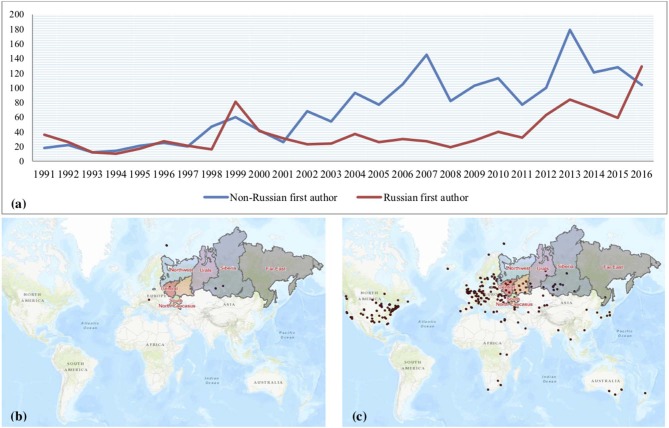
**(a)** Russia vs. non-Russia first authors over the study period, **(b)** location of first authors as of December 31, 1991, and **(c)** location of first authors as of December 31, 2016.

From 1991 to 2001 the numbers of Russian and non-Russian first authors were similar. Starting in 2002, the number of non-Russian first authors became three times more common than Russian first authors, and that trend continued over time, potentially indicating an increasing number of global collaborations. This is displayed in Figure 2 with a world map of first author locations in 1991 and 2016 for comparison.

**Figure 3:**
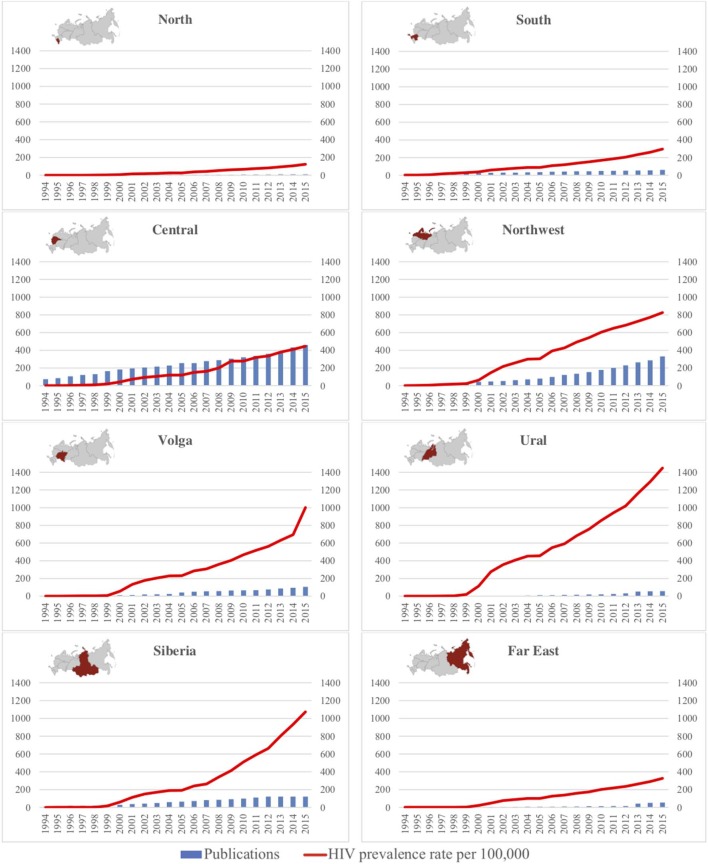
Temporal trends in publications and HIV prevalence per Russian federal district.

Within the Russian Federation there were 15 publications per million of population. Total productivity over time per district was plotted with HIV prevalence rate using available national epidemiologic data from the Russian Federation for the years 1994–2015 (Figure 3).

The most prolific federal districts for published research were Central and Northwest. Ural and Siberia had the highest HIV prevalence rates, though their published research productivity was comparatively much lower. The North, South and Far East federal districts showed a slower increase in HIV prevalence with similarly low numbers of published research.

**Figure 4:**
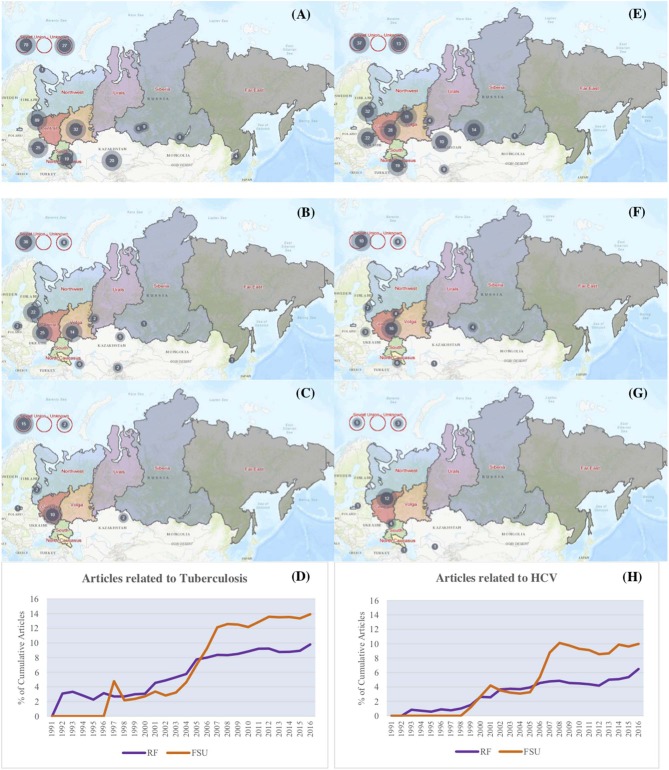
Number of research articles during the study period with a *Mycobacteria tuberculosis* research theme in 2016 **(A)**, 2005 **(B)**, and 2000 **(C)** or a Hepatitis C Virus research theme in 2016 **(E)**, 2005 **(F)**, and 2000 **(G)**. **(D,H)** show the percentage of articles with *Mycobacteria tuberculosis* and Hepatitis C Virus research themes, respectively, over time.

Given thematic analyses and corresponding to the priorities within the Russian Federation's state strategy to combat HIV and comorbidities, we evaluated trends in TB and HCV research themes over time. The percentage of articles per year related to both TB and HCV as a proportion of the total number of articles trended up in both the Russian Federation and countries of the former Soviet Union, yet this increase was significantly greater for countries of the former Soviet Union when compared to the Russian Federation (Figure 4). The trends in TB and HCV themed HIV research can also be visually depicted through use of the web-map, which shows the total articles related to TB and HCV by research location over time (Figure 4).

## Discussion

Our systematic geospatial review of HIV/AIDS related research in Eastern Europe/Central Asia and the Russian Federation revealed fewer than expected publications over a 25 year period by formal bibliometric analysis. Of the articles from the Russian Federation, there were 15 publications per million population, which is lower than what has been seen in other comparable bibliometric studies (16). Compared to the Russian Federation with its population of nearly 144 million, a bibliometric analysis of HIV/AIDS research in Lesotho, a country of <2 million people, there were nearly 1,280 articles published over the same time period (640 publications per million). A similar analysis of 27 European countries with currently much more limited HIV epidemics from 2002 to 2011 indexed a total of 90,564 articles with a median publication rate of 45 per million population (16). When evaluating specific research themes within the Russian Federation, there were even smaller numbers of publications addressing some of the largest issues pertinent to the HIV epidemic such as injection drug use, imprisonment, or the syndemics of HCV and TB.

There were also notable differences in research themes between the Russian Federation and countries of the former Soviet Union, the former weighted heavily toward themes of basic science. For example, the theme of virology was tagged in a third of all articles published in the Russian Federation. In contrast, psychosocial themes were less common, despite that substance use disorders, specifically IDU of opioids, play a major role in Russia's HIV epidemic and account for 48.8% of new HIV infections (2). The Russian Federation's border regions in southern Siberia are affected by large international opioid trade routes through Kazakhstan from Afghanistan, where the majority of the world's heroin and morphine are produced (5, 22). While IDU was a research theme in a quarter of articles from the Russian Federation, it was present in nearly half of those from countries of the former Soviet Union. This difference may reflect priorities in Russian health policy compared to other countries studied (23). For example, treatment options for PWID in the Russian Federation focus on detoxification while opioid substitution therapy, as recommended by the World Health Organization (23), remains legally prohibited. Of the 15 countries of the former Soviet Union, only the Russian Federation legally prohibits opioid substitution therapy and apart from Turkmenistan it is available in every other former Soviet country. Kyrgyzstan, Tajikistan, Moldova and Armenia also offer needle-syringe programs and opioid substitution therapy in prisons (10, 24–26).

Relatedly, a low proportion of all articles from the Russian Federation (3.5%) were tagged with the theme of prisoners/ incarceration. Yet the Russian Federation has the highest incarceration rate of any country in Eastern Europe (446 per 100,000), second in the world to the United States (27), and prisons are one of the main settings for the convergence of HIV, HCV and TB acquisition, all amplified by patterns of IDU (10). In the Russian Federation, the HIV prevalence in prisons is six times higher than the general population (10). Meanwhile increases in incarceration rates have been shown to account for increases in TB incidence, and TB outbreaks in the community have been linked to TB outbreaks in prisons (27). Co-infection of HIV and HCV among PWID in the Russian Federation is estimated to be as high as 90% (28). While children co-infected with HIV and HCV in the Russian Federation had the highest treatment rate (61%) for HCV of 4 other European countries in one report (29), this trend has not been noted in Russian adults. For instance, only 3% of patients with known HCV reported ever receiving HCV treatment in a cohort of PWID in St. Petersburg (11). Given the significant treatment success rate for HCV achieved with contemporary direct acting antiviral agents for HCV (30), comparable increases in HIV/HCV research output would have been expected. While we observed a proportional increase in HCV themed research in the Russian Federation over the bibliographic time period, the increase was more pronounced for countries of the former Soviet Union.

The Russian government's State Strategy goals include the development of new technologies for prevention, diagnosis and treatment of HIV with scientifically informed responses (7). There has been an early focus on HIV screening relative to the remaining cascade of HIV care, although current signs suggest a growing commitment to treatment as prevention efforts (3), including among people presenting for hepatitis or TB care (13, 31). Considering the staggering number of TB infections in the Russian Federation with increasing numbers of MDR-TB and extensively drug-resistant-TB (XDR-TB), indicating resistance to nearly all classes of TB therapy, there was a lower than expected proportion of articles with TB as a research theme (only 10.8% of Russian articles). There was no significant increase in TB themed research over time when compared to countries of the former Soviet Union. TB prevalence also varies among Russian federal districts and is 1.6 times more common in Siberia, and increasingly so among the population of PWID (5, 32). Importantly, we observed the most notable mismatches between scientific output and population adjusted estimates of HIV prevalence in the Siberian and neighboring Ural and Volga regions. Mirroring the separation of HIV and TB research in the Russian Federation, present Russian health care infrastructure favors the division of TB and HIV services. For example, TB physicians cannot directly prescribe antiretroviral therapy. It must be approved by committee and distributed through centralized HIV treatment facilities (32). Our findings suggest that especially in regions such as Volga, Ural and Siberia, expanded research efforts to study access and retention in care for PLWH and TB or PWID are needed to fill critical knowledge gaps.

Two other major high risk populations for HIV acquisition and transmission are MSM and sex workers. There was a statistically significant difference in total number of publications addressing sex workers between the Russian Federation and countries of the former Soviet Union (4.8 vs. 9.3%, *p* = <0·001) with a similar pattern for MSM (4.9 vs. 7.3%, *p* = 0.018). Limited epidemiologic data in the Russian Federation estimated rates of HIV acquisition due to MSM to be 4–6%, but a recent respondent driven sampling study in Moscow showed that of MSM tested for HIV there was a prevalence of >15% (33). Together this suggests that the true magnitude of MSM as an HIV risk group is likely underestimated. The majority of MSM related publications originated from the Central and Northwest districts (78%). While these districts are the most populated regions in the Russian Federation, the small number of publications in the remainder of the Federation is noteworthy.

Our approach of macro bibliometric analysis carries inherent limitations. Firstly, articles were tagged with research themes based on abstract review alone and may therefore have been incomplete or subject to misclassification. We suspect this form of classification bias was small in magnitude and without directionality, as abstracts were reviewed by committee. Importantly, the literature search likely did not capture all output in the Russian Federation or countries of the former Soviet Union. The MeSH term “Soviet Union” was an umbrella term for seven of the 15 countries (Armenia, Georgia, Kazakhstan, Kyrgyzstan, Moldova, Ukraine and Uzbekistan). Countries that were not included in the umbrella were Estonia, Latvia, Lithuania, Azerbaijan, Tajikistan, Uzbekistan, and Belarus. Thus, while many articles regarding the eight countries not included within the umbrella term were still captured because “Soviet Union” was found in the title or abstract, this may partly explain the observed frequency of articles from Ukraine. Output that was not published in publicly available sources, as is common for doctoral theses, “gray” literature, or working reports, or even that which was published in smaller journals that were not indexed in the major databases we utilized would also be missed. While the majority of research from countries of the former Soviet Union will at least have been referenced in English, if research was exclusively referenced in a database that was not English or Russian language, it may not have been included. Accessing research that was not in databases would have required extensive onsite participation and authorization from local universities, ministries of health and other repositories that would have been beyond the scope of this review and would have been subject to retrieval biases. Additionally, the comparison of research output relative to the Russian Federation regional HIV prevalence was dependent upon regional reports that did not account for deaths among people living with HIV or comparative proportions tested negative for HIV. True prevalence or incidence could not accurately be recorded. A small number of articles may have been represented as multiple data points in the analysis because of links to multiple countries or cities within the Russian Federation. Lastly, given the number and diversity of countries within the former Soviet states and the potential underrepresentation of individual countries throughout the study period given our search strategy, the bulk of secondary analyses and policy related discussion was restricted to the Russian Federation.

## Conclusion

This geospatial bibliometric review of HIV/AIDS related scientific literature in the Russian Federation and countries of the former Soviet Union shows a lower than expected publication rate in the Russian Federation in response to a massive and growing HIV epidemic. Differences in research themes between the Russian Federation and countries of the former Soviet Union and lagging publication rates among Russian federal districts with high HIV prevalence may indicate opportunities for new research priorities to guide future HIV interventions, in conjunction with those currently proposed by the Russian government. Our findings suggest such focus to include the federal districts of Volga, Ural and Siberia with expansion of interventional research in HIV-related TB and viral hepatitis, and among higher risk populations, such as PWID. An interactive web-map displaying these findings was successfully developed and is now available as a tool for researchers and policymakers.

## Author Contributions

HP, AK, AT, SV, and MG performed data collection and analysis. HP, AK, SH, OO, GL, RD, EZ, and YP contributed to web-map development. MG and SH wrote the manuscript. YP, EZ, OO, RD, and SH provided edits to the manuscript.

### Conflict of Interest

The authors declare that the research was conducted in the absence of any commercial or financial relationships that could be construed as a potential conflict of interest.

## Abbreviations

HIV: Human Immunodeficiency Virus
RF: the Russian Federation
FSU: Countries of the Former Soviet Union
HIV: Human Immunodeficiency Virus
PWID: people who inject drugs
MSM: men who have sex with men
IDU: Injection Drug Use
AIDS: Acquired Immunodeficiency Syndrome
HCV: hepatitis C virus
TB: Mycobacterium tuberculosis
MDR-TB: Multidrug resistant *Mycobacterium tuberculosis*.

## References

[1] Unaids. AIDS by the Numbers, AIDS is not Over, But it Can Be. (2020). Available online at: http://www.unaids.org/sites/default/files/media_asset/AIDS-by-the-numbers-2016_en.pdf (accessed April 19, 2018).

[2] Federal Scientific for the Prevention and Combat of AIDS of the Public Office of the Central Scientific Research Institute Rospotrebnadzor Spravka, VICH infektsiya v Rossiiskoi Federatsiy na 30 iyunia 2016 goda”? Reference on HIV infection in the Russian Federation as of June 30, 2016”. Perm Krai Public Heal Off Perm Krai Cent Prev Combat AIDS Infect.

[3] King EJ Civil society and health. In: Greer SL Wismar M Pastorino G Kosinska M, editors. Civil Society and Health: Contributions and Potential. Copenhagen: World Health Organization (2017). Sections 8.1–8.4 29064647

[4] Pratt GF. A decade of AIDS literature. Bull Med Libr Assoc. (1992) 80:380–1. Available online at: https://core.ac.uk/download/pdf/46714697.pdf 1422512 PMC225705

[5] Beyrer C Wirtz AL O'Hara G Léon N Kazatchkine M. The expanding epidemic of HIV-1 in the Russian Federation. PLoS Med. (2017) 14:e1002462. 10.1371/journal.pmed.1002462 29182631 PMC5705067

[6] Global Burden of Disease Profile Russian Federation. (2018). Available online at: http://www.healthdata.org/russia (accessed February 19, 2019).

[7] Russian Government Approving the State Strategy to Combat the Spread of HIV in Russia Through 2020 and Beyond. (2016). Available online at: http://government.ru/en/docs/24983/ (accessed April 19, 2018).

[8] Fierer DS Dieterich DT Fiel MI Branch AD Marks KM Fusco DN. Rapid progression to decompensated cirrhosis, liver transplant, and death in HIV-infected men after primary hepatitis C virus infection. Clin Infect Dis. (2013) 56:1038–43. 10.1093/cid/cis1206 23264364 PMC3588118

[9] Nelson PK Mathers BM Cowie B Hagan H Des Jarlais D Horyniak D. Global epidemiology of hepatitis B and hepatitis C in people who inject drugs: results of systematic reviews. Lancet. (2011) 378:571–83. 10.1016/S0140-6736(11)61097-0 21802134 PMC3285467

[10] Altice FL Azbel L Stone J Brooks-Pollock E Smyrnov P Dvoriak S. The perfect storm: incarceration and the high-risk environment perpetuating transmission of HIV, hepatitis C virus, and tuberculosis in Eastern Europe and Central Asia. Lancet. (2016) 388:1228–48. 10.1016/S0140-6736(16)30856-X 27427455 PMC5087988

[11] Tsui JI Ko SC Krupitsky E Lioznov D Chaisson CE Gnatienko N. Insights on the Russian HCV care cascade: minimal HCV treatment for HIV/HCV co-infected PWID in St. Petersburg. Hepatol Med Policy. (2016) 1:13. 10.1186/s41124-016-0020-x 28217368 PMC5313079

[12] Yablonskii PK Vizel AA Galkin VB Shulgina MV. Tuberculosis in Russia. Its history and its status today. Am J Respir Crit Care Med. (2015) 191:372–6. 10.1164/rccm.201305-0926OE 25679104

[13] Keshavjee S Yedilbayev A Sweeney C The Sputnik Initiative: Patient-Centered Accompaniment for Tuberculosis in Russia | Partners in Health. (2014). Available online at: https://www.pih.org/practitioner-resource/the-sputnik-initiative-patient-centered-accompaniment-for-tuberculosis-in-r (accessed July 5, 2018).

[14] TB/HIV coinfection (2018). Available online at: http://www.euro.who.int/en/health-topics/communicable-diseases/tuberculosis/data-and-statistics/tbhiv-coinfection (accessed July 5, 2018).

[15] Post FA Grint D Werlinrud AM Panteleev A Riekstina V Malashenkov EA. Multi-drug-resistant tuberculosis in HIV positive patients in Eastern Europe. J Infect. (2014) 68:259–63. 10.1016/j.jinf.2013.09.034 24247067

[16] Uusküla A Toompere K Laisaar KT Rosenthal M Pürjer ML Knellwolf A. HIV research productivity and structural factors associated with HIV research output in European Union countries: a bibliometric analysis. BMJ Open. (2015) 5:e006591. 10.1136/bmjopen-2014-006591 25649212 PMC4322208

[17] Fajardo-Ortiz D Lopez-Cervantes M Duran L Dumontier M Lara M Ochoa H. The emergence and evolution of the research fronts in HIV/AIDS research. PLoS ONE. (2017) 12:e0178293. 10.1371/journal.pone.0178293 28542584 PMC5444800

[18] Mugomeri E Bekele BS Mafaesa M Maibvise C Tarirai C Aiyuk SE. A 30-year bibliometric analysis of research coverage on HIV and AIDS in Lesotho. Health Res Policy Syst. (2017) 15:21. 10.1186/s12961-017-0183-y 28320397 PMC5360085

[19] Uthman OA. Pattern and determinants of HIV research productivity in sub-Saharan Africa: bibliometric analysis of 1981 to 2009 PubMed papers. BMC Infect Dis. (2010) 10:47. 10.1186/1471-2334-10-47 20205717 PMC2841182

[20] Falagas ME Bliziotis IA Kondilis B Soteriades ES. Eighteen years of research on AIDS: contribution of and collaborations between different world regions. AIDS Res Hum Retroviruses. (2006) 22:1199–205. 10.1089/aid.2006.22.1199 17209761

[21] Bai J Li W Huang Y-M Guo Y. Bibliometric study of research and development for neglected diseases in the BRICS. Infect Dis Poverty. (2016) 5:89. 10.1186/s40249-016-0182-1 27595987 PMC5011792

[22] United Nations Office on Drugs and Crime Drug Trafficking. (2018). Available online at: http://www.unodc.org/unodc/en/drug-trafficking/index.html (accessed July 5, 2018).

[23] World Prison Brief (2016). Available online at: http://www.prisonstudies.org/world-prison-brief-data (accessed April 18, 2018).

[24] Larney S Peacock A Leung J Colledge S Hickman M Vickerman P. Global, regional, and country-level coverage of interventions to prevent and manage HIV and hepatitis C among people who inject drugs: a systematic review. Lancet Glob Heal. (2017) 5:e1208–20. 10.1016/S2214-109X(17)30373-X 29074410 PMC5683737

[25] Parsons D Burrows D Bolotbaeva A. Advocating for opioid substitution therapy in Central Asia: much still to be done. Int J Drug Policy. (2014) 25:1174–7. 10.1016/j.drugpo.2014.01.004 24680628

[26] Chingin A Fedorova O Turkmenistan Drug Situation and Drug Policy Pompidou Group of the Council of Europe Co-operation Group to Combat Drug Abuse and Illicit Trafficking in Drugs. 2014. Available online at: https://rm.coe.int/drug-situation-and-drug-policy-by-alex-chingin-and-olga-fedorova-decem/168075f300 (accessed August 6, 2018).

[27] Stuckler D Basu S McKee M King L. Mass incarceration can explain population increases in TB and multidrug-resistant TB in European and central Asian countries. Proc Natl Acad Sci USA. (2008) 105:13280–5. 10.1073/pnas.0801200105 18728189 PMC2533181

[28] Report of the Global Commission on Drug Policy The Negative Impact of the War on Drugs on Public Health: The Hidden Hepatitis C Epidemic. (2013). Available online at: www.arud.ch (accessed July 5, 2018).

[29] Turkova A Giacomet V Goetghebuer T Mikoenko M Nicolini LA Noguera-Julian A. HCV treatment in children and young adults with HIV/HCV co-infection in Europe. J virus Erad. (2015) 1:179–84. 27482410 10.1016/S2055-6640(20)30504-5 PMC4946737

[30] Gutierrez JA Lawitz EJ Poordad F. Interferon-free, direct-acting antiviral therapy for chronic hepatitis C. J Viral Hepat. (2015) 22:861–70. 10.1111/jvh.12422 26083155

[31] Ogarkov OB Ebers A Zhdanova S Moiseeva E Koshcheyev ME Zorkaltseva E. Administrative interventions associated with increased initiation on antiretroviral therapy in Irkutsk, Siberia. Public Heal Action. (2016) 6:252–4. 10.5588/pha.16.0050 28123963 PMC5176050

[32] Heysell SK Ogarkov OB Zhdanova S Zorkaltseva E Shugaeva S Gratz J. Undertreated HIV and drug-resistant tuberculosis at a referral hospital in Irkutsk, Siberia. Int J Tuberc Lung Dis. (2016) 20:187–92. 10.5588/ijtld.14.0961 26792470 PMC4863947

[33] Wirtz AL Zelaya CE Latkin C Peryshkina A Galai N Mogilniy V. The HIV care continuum among men who have sex with men in Moscow, Russia: a cross-sectional study of infection awareness and engagement in care. Sex Transm Infect. (2016) 92:161–7. 10.1136/sextrans-2015-052076 26297721 PMC4889127

