# Supplementary material for: A Geospatial Bibliometric Review of the HIV/AIDS Epidemic in the Russian Federation
Source: Front Public Health. 2020 Apr 2;8:75. doi: 10.3389/fpubh.2020.00075 (PMC7145403; doi:10.3389/fpubh.2020.00075)
Supplement: Supplementary file 1 [file Table_1.docx]

|  | **Former Soviet Union Countries** | | | | | | | | | | | | | | | |
| --- | --- | --- | --- | --- | --- | --- | --- | --- | --- | --- | --- | --- | --- | --- | --- | --- |
|  | Total | Armenia | Azerbaijan | Belarus | Estonia | Georgia | Kazakhstan | Kyrgyzstan | Latvia | Lithuania | Moldova | Tajikistan | Turkmenistan | Ukraine | Uzbekistan | FSU - Unknown |
| *Number of Articles* | 712 | 15 | 11 | 45 | 102 | 63 | 50 | 29 | 30 | 29 | 20 | 23 | 6 | 208 | 30 | 51 |
| *Topics* | *(% of Total)* | *2.1* | *1.5* | *6.3* | *14.3* | *8.8* | *7* | *4.1* | *4.2* | *4.1* | *2.8* | *3.2* | *0.8* | *29.2* | *4.2* | *7.2* |
| *Injection Drug Use* | 346 | 5 | 5 | 19 | 45 | 31 | 24 | 17 | 11 | 9 | 11 | 14 | 3 | 115 | 13 | 24 |
| *Virology* | 107 | 0 | 2 | 23 | 16 | 4 | 10 | 3 | 4 | 5 | 6 | 1 | 0 | 19 | 7 | 7 |
| *STIs* | 163 | 7 | 1 | 4 | 29 | 14 | 14 | 5 | 5 | 8 | 5 | 5 | 4 | 30 | 13 | 19 |
| *Genetics/Genomics* | 117 | 0 | 1 | 18 | 17 | 8 | 8 | 4 | 6 | 5 | 6 | 2 | 0 | 28 | 8 | 6 |
| *Prevention* | 151 | 3 | 1 | 4 | 16 | 15 | 13 | 5 | 4 | 6 | 5 | 4 | 1 | 52 | 8 | 14 |
| *Antiretroviral Therapy* | 87 | 1 | 1 | 6 | 11 | 10 | 4 | 2 | 3 | 0 | 2 | 3 | 0 | 37 | 4 | 3 |
| *Policy* | 132 | 5 | 0 | 4 | 6 | 7 | 8 | 12 | 2 | 4 | 4 | 7 | 4 | 52 | 7 | 10 |
| *Infants Children Adolescents* | 118 | 4 | 4 | 4 | 11 | 14 | 7 | 5 | 2 | 3 | 3 | 3 | 2 | 41 | 10 | 5 |
| *Tuberculosis* | 99 | 0 | 1 | 4 | 17 | 10 | 9 | 5 | 11 | 2 | 3 | 3 | 1 | 22 | 3 | 8 |
| *Substance Use Disorder* | 106 | 4 | 3 | 6 | 12 | 7 | 6 | 4 | 2 | 1 | 3 | 6 | 2 | 34 | 8 | 8 |
| *Opportunistic Infections* | 91 | 3 | 3 | 5 | 11 | 9 | 6 | 5 | 6 | 5 | 2 | 2 | 2 | 15 | 6 | 11 |
| *Access to Care* | 98 | 2 | 1 | 4 | 8 | 4 | 6 | 3 | 3 | 2 | 4 | 3 | 3 | 42 | 5 | 8 |
| *HCV Co-infection* | 71 | 0 | 3 | 4 | 8 | 16 | 7 | 1 | 3 | 6 | 3 | 4 | 0 | 8 | 4 | 4 |
| *Stigma* | 62 | 1 | 2 | 0 | 7 | 7 | 3 | 2 | 1 | 7 | 0 | 3 | 2 | 23 | 2 | 2 |
| *Sex Workers* | 66 | 6 | 1 | 3 | 6 | 2 | 3 | 5 | 2 | 2 | 3 | 1 | 3 | 18 | 7 | 4 |
| *Alcohol* | 28 | 3 | 0 | 1 | 6 | 2 | 2 | 1 | 1 | 0 | 1 | 0 | 0 | 8 | 0 | 3 |
| *MSM* | 52 | 1 | 2 | 2 | 11 | 4 | 5 | 1 | 3 | 5 | 2 | 1 | 2 | 5 | 2 | 6 |
| *Prisoners/Incarceration* | 38 | 0 | 2 | 2 | 3 | 3 | 3 | 0 | 2 | 0 | 0 | 2 | 1 | 15 | 0 | 5 |
| *Adherence* | 23 | 0 | 0 | 0 | 6 | 2 | 2 | 0 | 3 | 2 | 0 | 0 | 0 | 7 | 1 | 0 |
| *Psychiatric Illness* | 6 | 0 | 1 | 0 | 0 | 0 | 0 | 0 | 0 | 0 | 0 | 0 | 0 | 3 | 0 | 2 |
| *PrEP* | 11 | 0 | 0 | 1 | 0 | 2 | 0 | 0 | 0 | 1 | 0 | 0 | 0 | 6 | 0 | 1 |
| *Retention in Care* | 5 | 0 | 0 | 0 | 0 | 1 | 0 | 0 | 0 | 0 | 0 | 0 | 0 | 3 | 0 | 1 |
| *Coping/Spirituality* | 4 | 0 | 0 | 0 | 0 | 0 | 0 | 0 | 0 | 1 | 0 | 0 | 0 | 3 | 0 | 0 |
| *Disclosure* | 6 | 1 | 0 | 0 | 2 | 0 | 0 | 0 | 1 | 1 | 0 | 0 | 1 | 0 | 0 | 0 |
| *Mobile Health* | 1 | 0 | 0 | 0 | 0 | 0 | 0 | 0 | 0 | 0 | 0 | 0 | 0 | 1 | 0 | 0 |

Research theme order based on total dataset.

***Supplemental Table 1.* Quantity of research articles from the countries of the former Soviet Union separated by research theme from 1991 – 2016.**
