# Supplementary material for: A Geospatial Bibliometric Review of the HIV/AIDS Epidemic in the Russian Federation
Source: Front Public Health. 2020 Apr 2;8:75. doi: 10.3389/fpubh.2020.00075 (PMC7145403; doi:10.3389/fpubh.2020.00075)
Supplement: Supplementary file 2 [file Image_1.pdf]

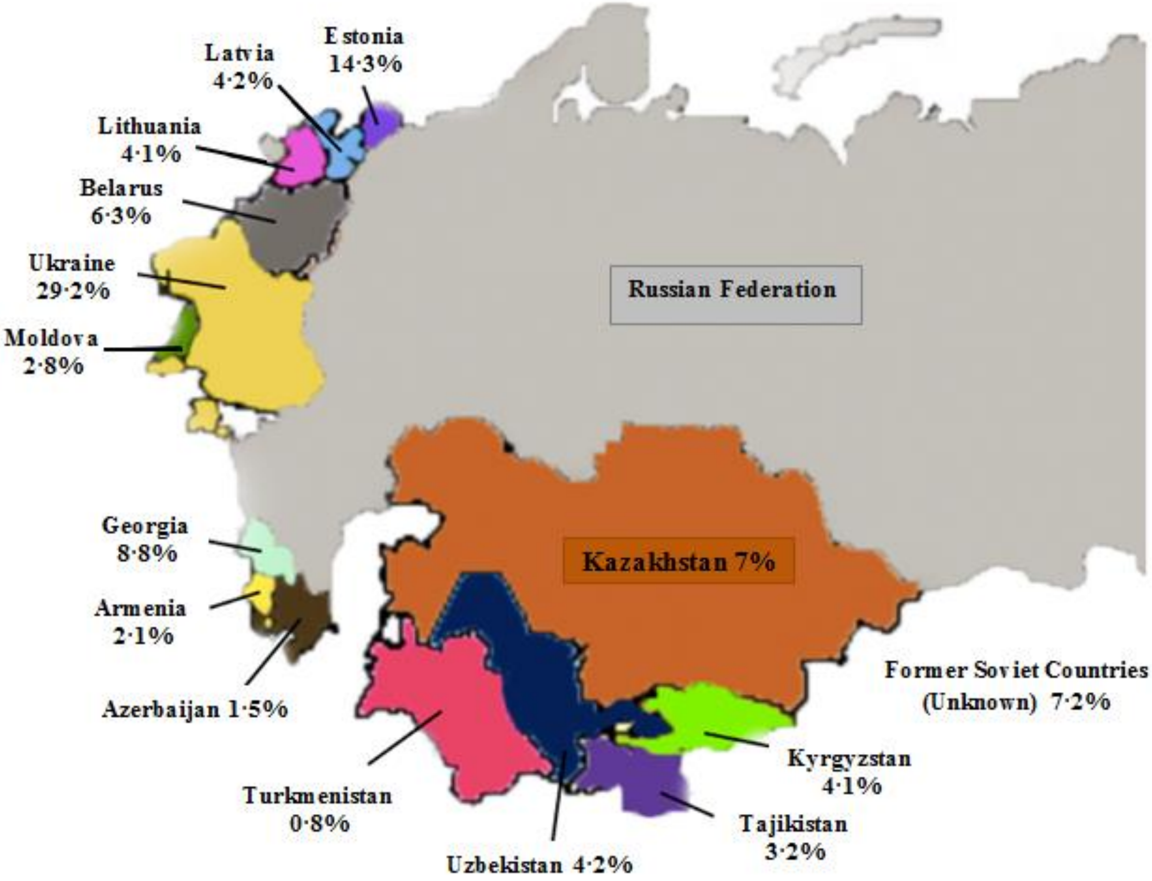

Supplemental Figure 1. **Map of the Countries of the Former Soviet Union with percentages of the total articles published by country.**
